# Dielectric properties of a bisimidazolium salt with dodecyl sulfate anion doped with carbon nanotubes

**DOI:** 10.3762/bjnano.9.19

**Published:** 2018-01-16

**Authors:** Doina Manaila Maximean, Viorel Cîrcu, Constantin Paul Ganea

**Affiliations:** 1 University Politehnica of Bucharest, Department of Physics, 313 Spl. Independentei, 060042, Bucharest, Romania; 2 Department of Inorganic Chemistry, University of Bucharest, 23 Dumbrava Rosie st, sector 2, Bucharest 020464, Romania; 3 National Institute of Materials Physics, POBox MG 07, 077125 Magurele, Romania

**Keywords:** activation energy, carbon nanotubes, dielectric spectroscopy, ionic liquid crystal, relaxation time

## Abstract

A new bisimidazolium salt with dodecyl sulfate as counterion has been designed and prepared. This salt shows a SmA phase that is stable at room temperature. The new ionic liquid crystal (ILC) was characterized by ^1^H NMR, ^13^C NMR and IR spectroscopy. Its liquid crystalline properties were analyzed by polarizing optical microscopy (POM), differential scanning calorimetry (DSC) and powder X-ray diffraction (XRD) studies. The dielectric spectra of the ILC doped with different concentrations of carbon nanotubes (CNT) were recorded over a wide frequency and temperature range of 10^−1^ to 10^7^ Hz and 293–338 K, respectively. The values of the activation energy were found in the range of 0.46–0.61 eV; the characteristic time was obtained by fitting the spectra of the dielectric loss with the Havriliak–Negami functions. As a result of doping the ILC with CNT, the electric conductivity increases significantly. Ionic conductivity is dominant and it was indirectly observed through the electrode polarization (EP) effect. The very high dielectric permittivity values and the decrease of the electric conductivity at low frequencies confirm the presence of EP.

## Introduction

Ionic liquid crystals (ILCs) represent a very appealing class of materials that has found various recent applications in dye-sensitized solar cells, battery materials, electrochemical sensors or energy storage devices. Their interesting properties result from the combination of liquid crystal (LC) and ionic liquid (IL) properties. The recent progress and development in the field of ILCs were reviewed in several publications [1–3]. There is a growing interest in ILs based on imidazolium cations. Particular attention is paid to the flexibly or rigidly linked bisimidazolium salts, the so-called gemini ILs. Gemini ILs can show interesting surfactant and liquid crystalline properties [4–12] and such salts were used in many applications, ranging from catalysis [13] to biological [14–16] or biochemical applications [17–19]. Recently, we have shown that it is possible to exchange the smaller anions (Br^−^) with alkyl sulfate ions to yield new ILCs based on bisimidazolium salts with flexible methylene spacer and long alkyl tails [20]. The LC properties are influenced, in the order of impact, by spacer length, alkyl tail length and, finally, by the length of alkyl chains attached to the sulfate groups [21]. Furthermore, the liquid crystalline compounds with alkyl sulfate anions have lower melting and clearing points. Hence, there is the interest to design such materials for further electro-optical applications [22–32].

**Figure 1:**
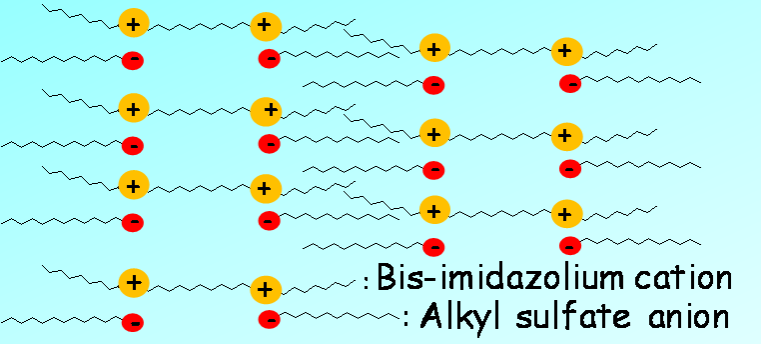
Schematic representation of the bilayer interdigitated SmA phase of the ILC based on bisimidazolium salts with alkyl sulfate ion.

Carbon nanotubes (CNTs) and nanoparticles were also dispersed in LCs [33–36]. It is well-known that the order of the LCs can be imposed on the CNTs in such a way that the alignment axis of the CNTs is driven by the LC reorientation controlled by an electric field [37]. The concentration and the spatial distribution of charges in the LC matrix will be affected by the presence of CNTs and, hence, the conductivity will be changed [38–44].

There are studies dedicated to the dielectric spectroscopy (DS) of ILCs doped with CNT [45]. In the present work we studied the effect of added CNTs on the dielectric properties of a new ILC based on a bisimidazolium salt with a dodecyl sulfate ion. The dielectric spectra of pure and CNT-doped ILC were recorded in the frequency range from 10^−1^ to 10^7^ Hz and in the temperature range from 293 to 338 K corresponding to the different phases of the ILC (mesophase and isotropic state). The values of the permittivity, dielectric loss and conductivity were deduced from the dielectric studies. The activation energy was calculated by employing the Vogel–Fulcher–Tammann law while the characteristic time was obtained by fitting the spectra of the dielectric loss with the Havriliak–Negami functions.

## Results and Discussion

### Synthesis of the bisimidazolium salt

**Scheme 1:**
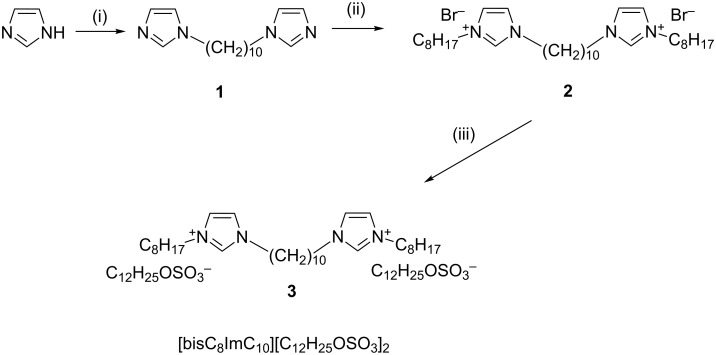
Synthesis of bisimidazolium salt with dodecyl sulfate anion: (i) Br(CH_2_)_10_Br, NaH; (ii) C_8_H_17_Br, acetonitrile; (iii) C_12_H_25_OSO_3_Na, methanol/dichloromethane.

The preparation of new bisimidazolium salt with dodecyl sulfate anion employed in this study, along with the numbering scheme of intermediates, is presented in Scheme 1.

In the first step 1,1′-(1,10-hexanediyl)bisimidazole (**1**) was prepared starting from imidazole and 1,10-dibromodecane, as described by Bara and co-workers [7]. The bromide salt **2** was prepared by alkylation of **1** with 1-bromooctane in acetonitrile under reflux. The product was precipitated with ethyl ether, and purified by several recrystallizations from dichloromethane/ethyl ether. The metathesis reaction of the bromide anion with sodium dodecyl sulfate, C_12_H_25_OSO_3_Na, yielded the corresponding salt [bisC_8_ImC_10_][C_12_H_25_OSO_3_]_2_ (**3**). Compound **3** was characterized by several physico-chemical techniques, such as elemental analysis (C, H, N), IR, ^1^H and ^13^C NMR spectroscopy, supporting the proposed structure. The exchange of bromide anion with dodecyl sulfate ion was easily confirmed by IR spectroscopy. The IR spectrum of [bisC_8_ImC_10_][C_12_H_25_OSO_3_]_2_ shows a strong band at 1225 cm^−1^ assigned to the sulfate group. Moreover, the ^1^H and ^13^C NMR spectra give additional support for the exchange of bromide ions with dodecyl sulfate ions. The signals assigned to the three protons belonging to the two imidazolium rings are shifted upfield in the ^1^H NMR spectrum of [bisC_8_ImC_10_][C_12_H_25_OSO_3_]_2_ compared to their position in the ^1^H NMR of the bromide salt **2**. The most significant change was observed for the signal assigned to the proton adjacent to the two nitrogen atoms (Scheme 1). For the bromide salt **2** this signal is located at 10.42 ppm, while for the dodecyl sulfate salt [bisC_8_ImC_10_][C_12_H_25_OSO_3_]_2_ this signal is shifted to 9.58 ppm. It is well documented that the anion–cation interactions have a strong effect on the NMR chemical shifts of protons belonging to the imidazolium ring. The NMR signals are shifted downfield due to the presence of hydrogen-bonding interactions in imidazolium-based ILC systems [32,46–51].

### Polarized optical microscopy (POM)

**Figure 2:**
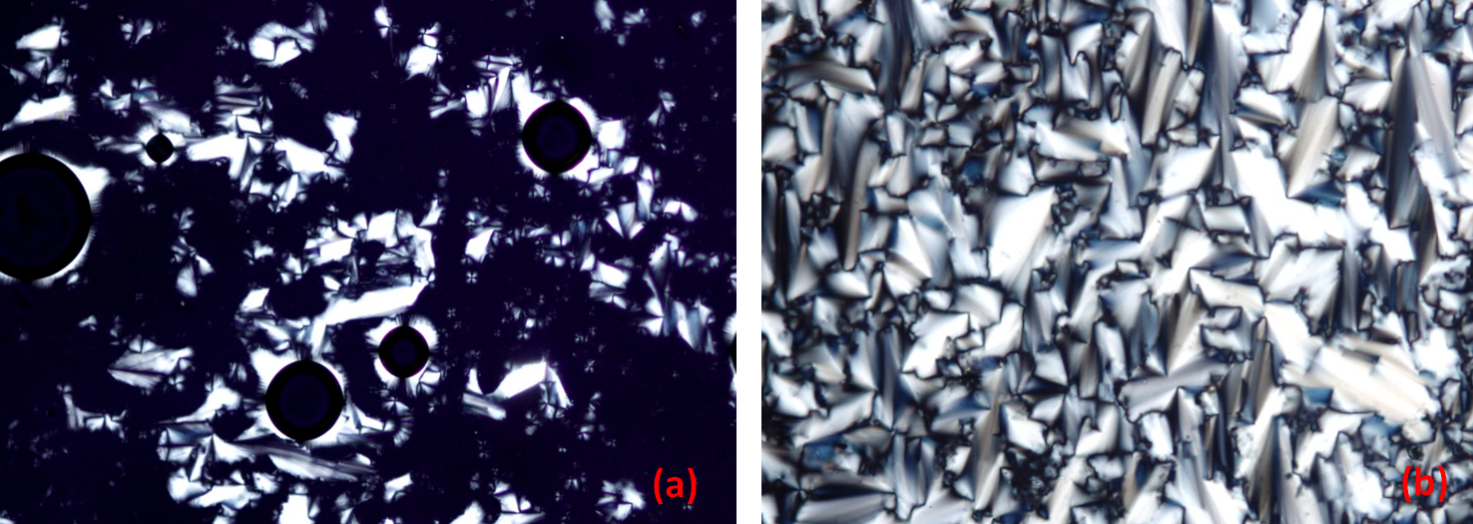
POM pictures of [bisC_8_ImC_10_][C_12_H_25_OSO_3_]_2_ on cooling from the isotropic state: at 318 K (a) and at 303 K (b).

The LC phase of [bisC_8_ImC_10_][C_12_H_25_OSO_3_]_2_ was determined based on the POM observations. Two different pictures of the textures developed on cooling from the isotropic state are shown in Figure 2. These observations were confirmed later by XRD studies. On cooling the sample from the isotropic state, typical fan-shape or focal conic textures together with several homeotropic areas were found, leading to an unambiguously assignment of a SmA phase. ILCs are well known to exhibit predominantly lamellar phases, with the SmA phase being the most common phase for such materials, in particular due to electrostatic interactions and ion–ion stacking in ILCs.

### Differential scanning calorimetry (DSC)

**Figure 3:**
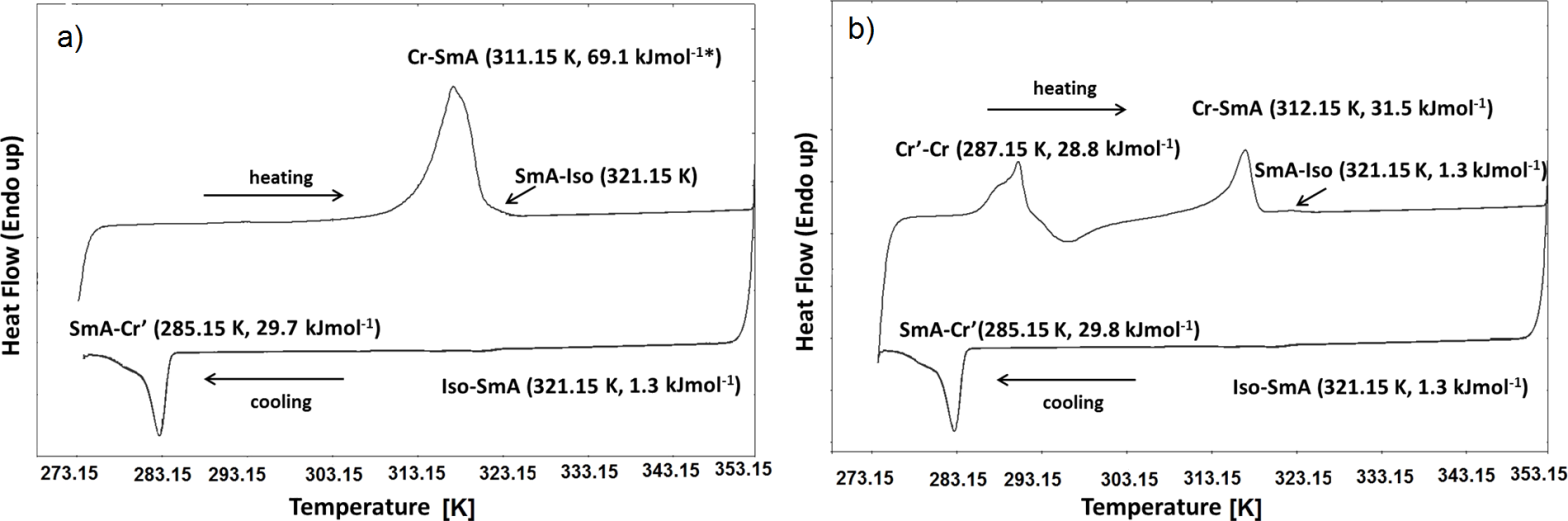
DSC traces for [bisC_8_ImC_10_][C_12_H_25_OSO_3_]_2_: (a) first and (b) second heating–cooling cycle recorded in the temperature range of 273–353 K with a scan rate of 10 K/min. Thermal parameters are given in brackets. The given enthalpy contains the combined values for the two transitions Cr–SmA and SmA–Iso.

The transitions and their corresponding temperatures together with the enthalpy values associated to these processes are presented in Figure 3. The transitions of [bisC_8_ImC_10_][C_12_H_25_OSO_3_]_2_ between mesophase and isotropic state observed by DSC are broad, with *T*_onset_ = 321.15 K. The temperature range of the liquid crystalline phase is limited to approx. 10 K during the heating run. The first heating run of the bisimidazolium salt shows a broad peak including the transition from the crystalline state to the LC phase and the following transition to the isotropic state, giving only the combined enthalpy of the two processes. However, POM observations clearly indicated that the two transformations are well separated. The following cooling run shows the Iso-SmA phase transition at 321.15 K followed by a second transition at 285.15 K, which was assigned to a transition from the LC phase to a different crystalline phase (Cr′). This second Cr′ phase is not thermodynamically stable at room temperature and it transitions to the first crystalline phase at 287.15 K during the subsequent heating run (in fact, it is a melting transition followed by the cold crystallization to the Cr phase). The following heating–cooling cycles are perfectly reproducible with the two transitions Cr–SmA and SmA–Iso well separated during the heating runs (Figure 3b). Importantly, POM observations show that on cooling from the isotropic state, the SmA phase is stable down to room temperature over time with a slow crystallization occurring over the course of hours based on POM observations. Obviously, the stability domain of the SmA phase is higher (ca. 36 K) during the cooling step, allowing for precise dielectric measurements in the LC phase.

### Powder X-ray diffraction measurements

**Figure 4:**
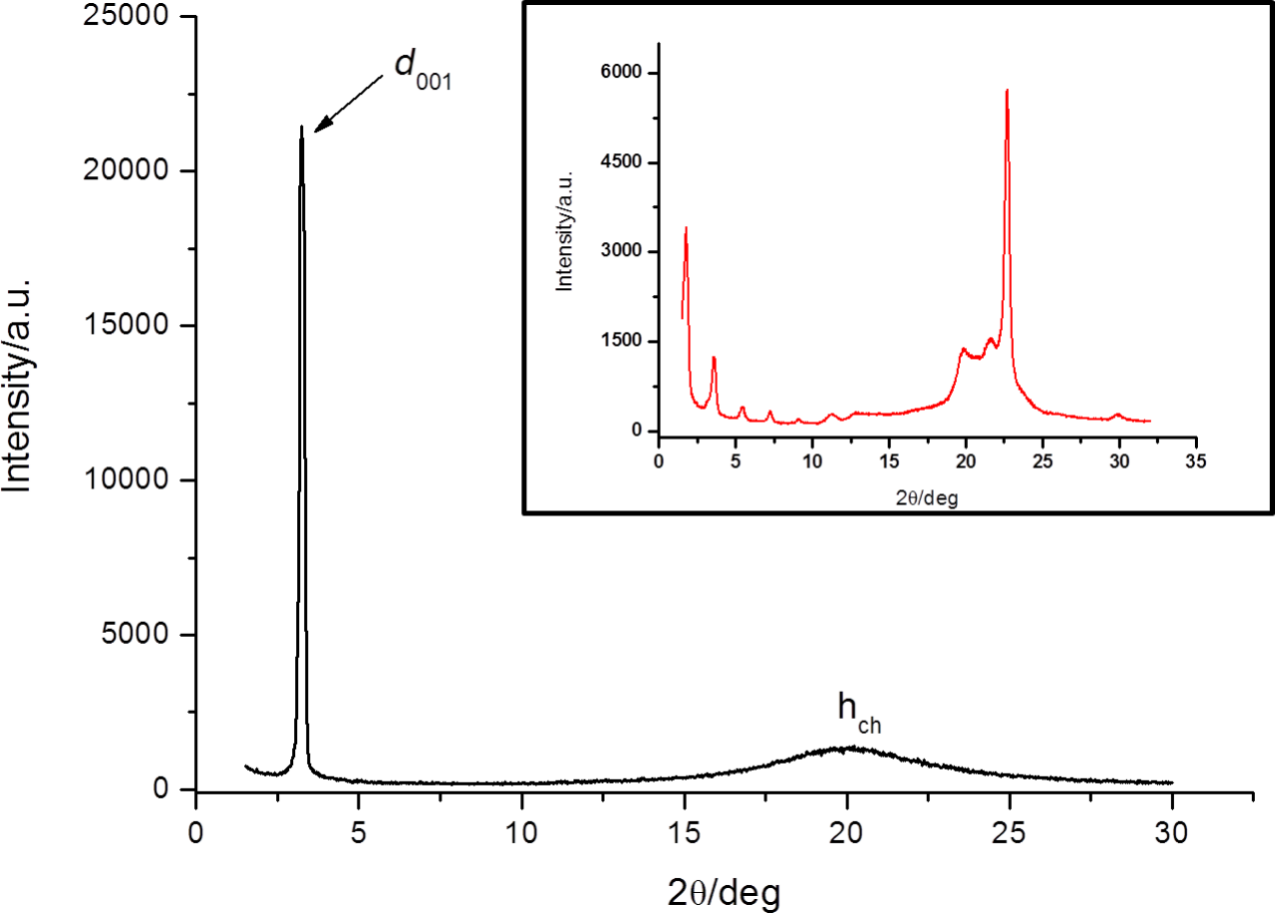
Powder XRD pattern of [bisC_8_ImC_10_][C_12_H_25_OSO_3_]_2_ recorded at 298 K, after cooling from the isotropic state. Inset: the XRD pattern recorded at 298 K in the crystalline state (prior to heating).

The nature of the LC phase was unequivocally confirmed by powder X-ray diffraction measurements. The XRD measurements were performed at 298 K after the sample was previously heated at 333 K to reach the isotropic state and then cooled down to room temperature in the mesophase temperature domain. The results are presented in Figure 4. The XRD pattern of [bisC_8_ImC_10_][C_12_H_25_OSO_3_]_2_ salt shows one sharp and intense diffraction peak in the small-angle region (27.3 Å) and a second broad peak at wide angles (around 4.5 Å). The first peak was assigned to the (001) reflection corresponding to a lamellar structure while the broad peak is due to the liquid-like order of the molten alkyl chains.

The results of a simple molecular calculation based on an all-trans extended model revealed a molecular length of about 39 Å. Indeed, by comparison of the experimental value of the layer thickness (27.3 Å) with the molecular length resulting from molecular calculations, it is justified to assume an interdigitated double layer structure for the SmA mesophase [4,20].

### Dielectric spectroscopy

The DS measurements were performed both for the pure ILC and for various mixtures of CNT-doped ILC (concentration 0.05% w/w and 0.5% w/w) in the frequency range from 10^−1^ to 10^7^ Hz. The temperature range was chosen in agreement with the DSC and the POM observations for the phase transitions, between 293 and 338 K.

**Figure 5:**
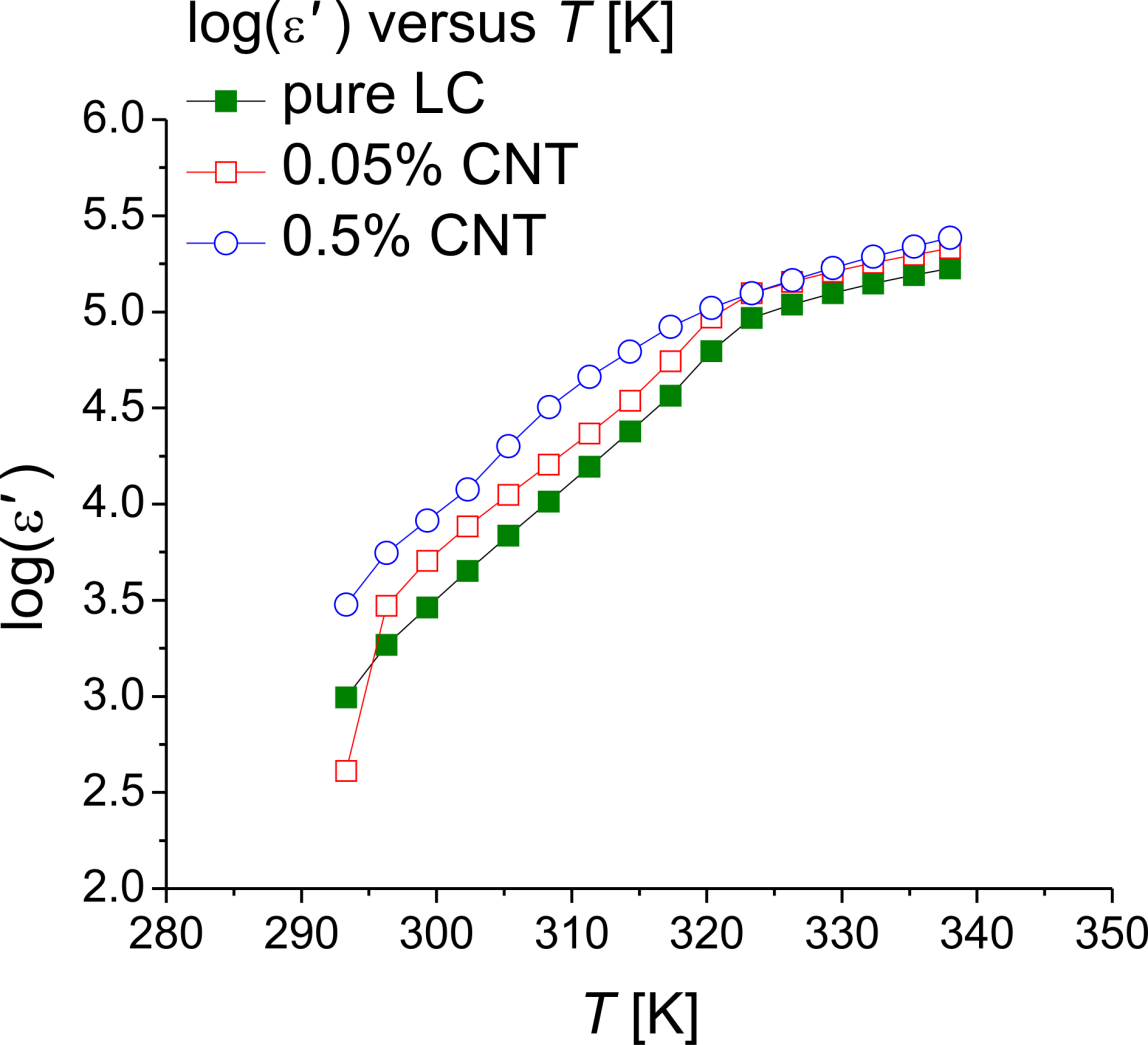
Permittivity as a function of the temperature (logarithmic scale) for pure ILC (green solid squares), ILC doped with 0.05% CNTs (red open squares) and ILC doped with 0.5% CNTs (blue open circles).

The logarithmic permittivity for the pure ILC and the CNT-doped ILC as a function of the temperature is presented in Figure 5. It was found that the permittivity has higher values for the CNT-doped ILC and, in the range of 320–338 K, the plots of the permittivity for the 0.05% CNT and 0.5% CNT concentrations overlap.

**Figure 6:**
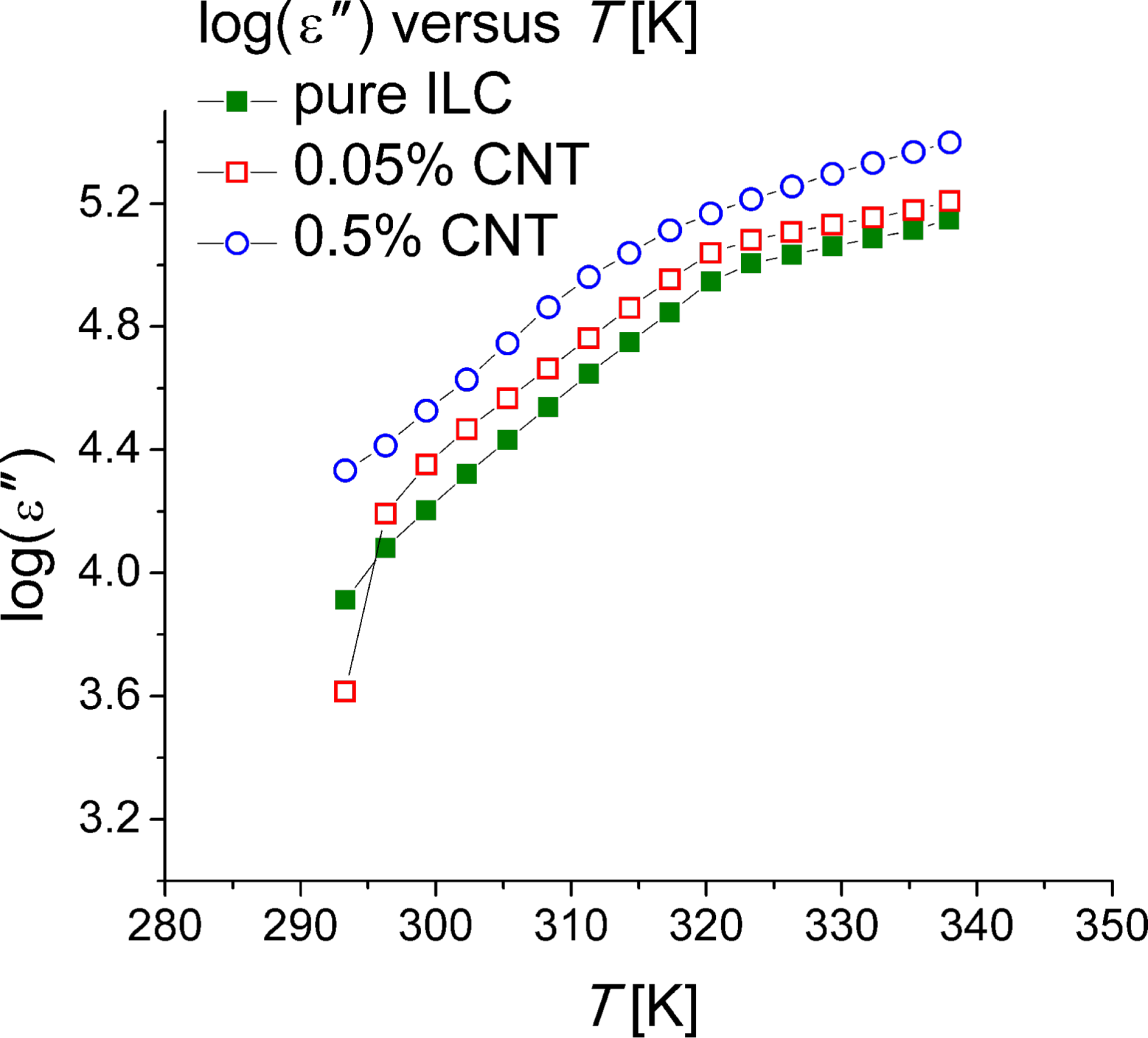
Dielectric loss as a function of the temperature (logarithmic scale) for pure ILC (green solid squares), ILC doped with 0.05% CNTs (red open squares) and ILC doped with 0.5% CNTs (blue open circles).

The temperature variation of the dielectric loss for the pure ILC and the CNT-doped ILC is shown in Figure 6. It was found that the dielectric loss increases with the temperature and with the CNT concentration.

The characteristic relaxation times were obtained by fitting the spectra of the dielectric loss with the Havriliak–Negami (HN) function [52]:

[1]
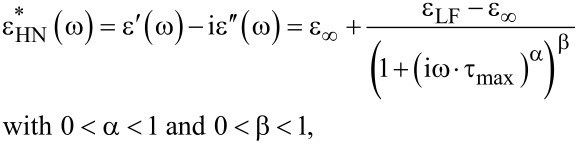


where ε′(ω) is the permittivity and ε″(ω) is the dielectric loss, ε_LF_ is the low-frequency (LF) permittivity and ε_∞_ is the permittivity in the high-frequency (HF) limit and τ_max_ is the characteristic relaxation time of the dielectric relaxation process.

The dependency τ_max_ = *f*(1/*T*) can be modeled using the Vogel–Fulcher–Tammann (VFT) law, as follows:

[2]
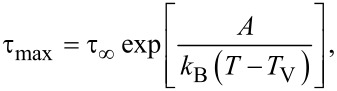


where *A* is a material constant, *k*_B_ is Boltzmann’s constant, *T* is the temperature, *T*_V_ is the Vogel temperature and τ_∞_ is a pre-exponential factor.

**Figure 7:**
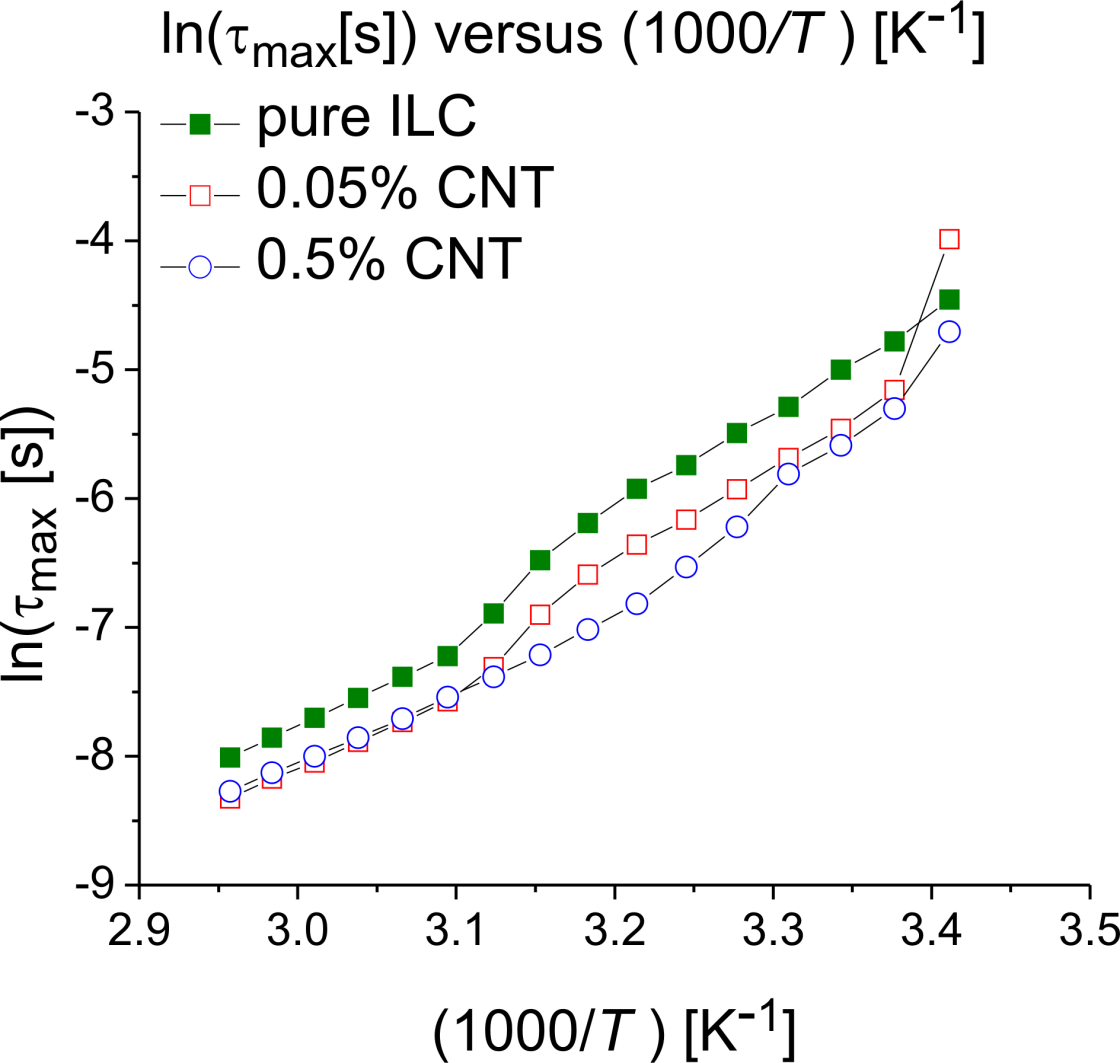
Relaxation time as function of the inverse temperature (logarithm scale) for pure ILC (green solid squares), ILC doped with 0.05% CNTs (red open squares) and ILC doped with 0.5% CNTs (blue open circles).

Figure 7 presents the characteristic relaxation time as a function of the inverse temperature for the pure ILC and the CNT-doped ILC. For the pure ILC and for the lower CNT doping concentrations, there are two slopes, attributed to the isotropic and the SmA phases. At the higher CNT concentration (0.5%) the transition isotropic–SmA could not be detected clearly, probably due to the very low energies involved in the transition. In the isotropic phase, the curves of the relaxation time for the 0.05% CNT and 0.5% CNT concentrations overlap, and for lower temperatures, the relaxation time decreases with the CNT concentration. The pure ILC has a relaxation time higher than the doped ILC in the temperature range of the mesophase.

Table 1 gives the values of the activation energy and the relaxation times in the corresponding temperature domains. The following observations can be made according to data presented in Table 1: a) the activation energy increases with CNT concentration; b) for the same CNT concentration, the activation energy is smaller in the isotropic phase (at higher temperatures).

**Table 1:** DS characteristic constants for pure and CNT-doped ILC.

no.	sample type	temperature domain [K]	activation energy, *E*_a_^a^ [eV]	relaxation time, τ_max_ [s]

1	pure ILC	296–317	0.61	5.500 × 10^−13^
		323–338	0.49	1.700 × 10^−11^
2	ILC doped with 0.05% CNT	293–317	0.59	1.169 × 10^−13^
		323–338	0.47	2.364 × 10^−11^
3	ILC doped with 0.5% CNT	309–338	0.46	3.105 × 10^−11^

^a^In these calculations, the Vogel temperature was considered zero in the initial fitting. Hence, the VFT equation approximates an Arrhenius-like expression, the material constant *A* being incorporated into the activation energy *E*_a_.

**Figure 8:**
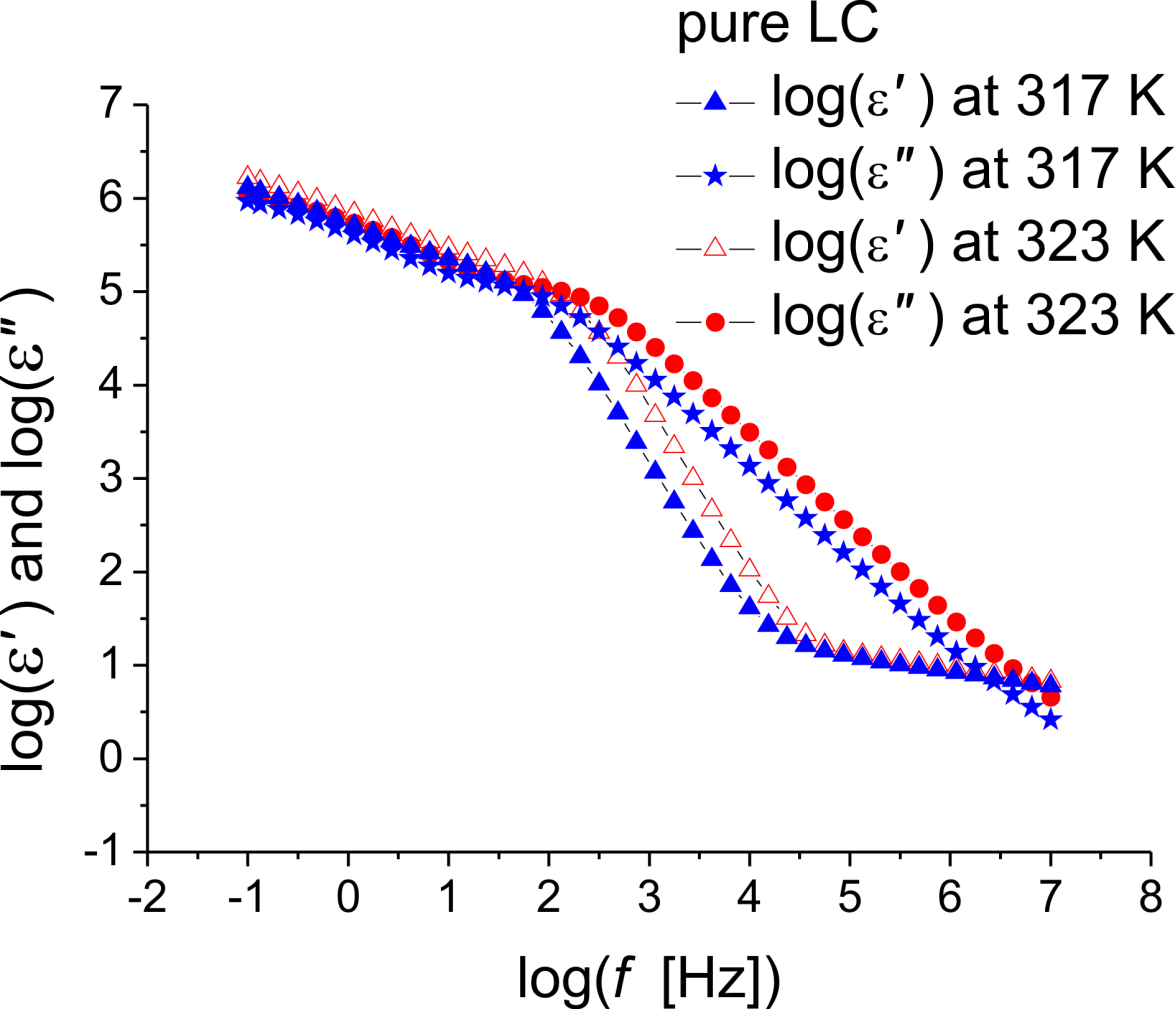
Permittivity and dielectric loss as functions of the frequency (logarithmic scale) for the pure ILC at two constant temperatures, 317 and 323 K.

**Figure 9:**
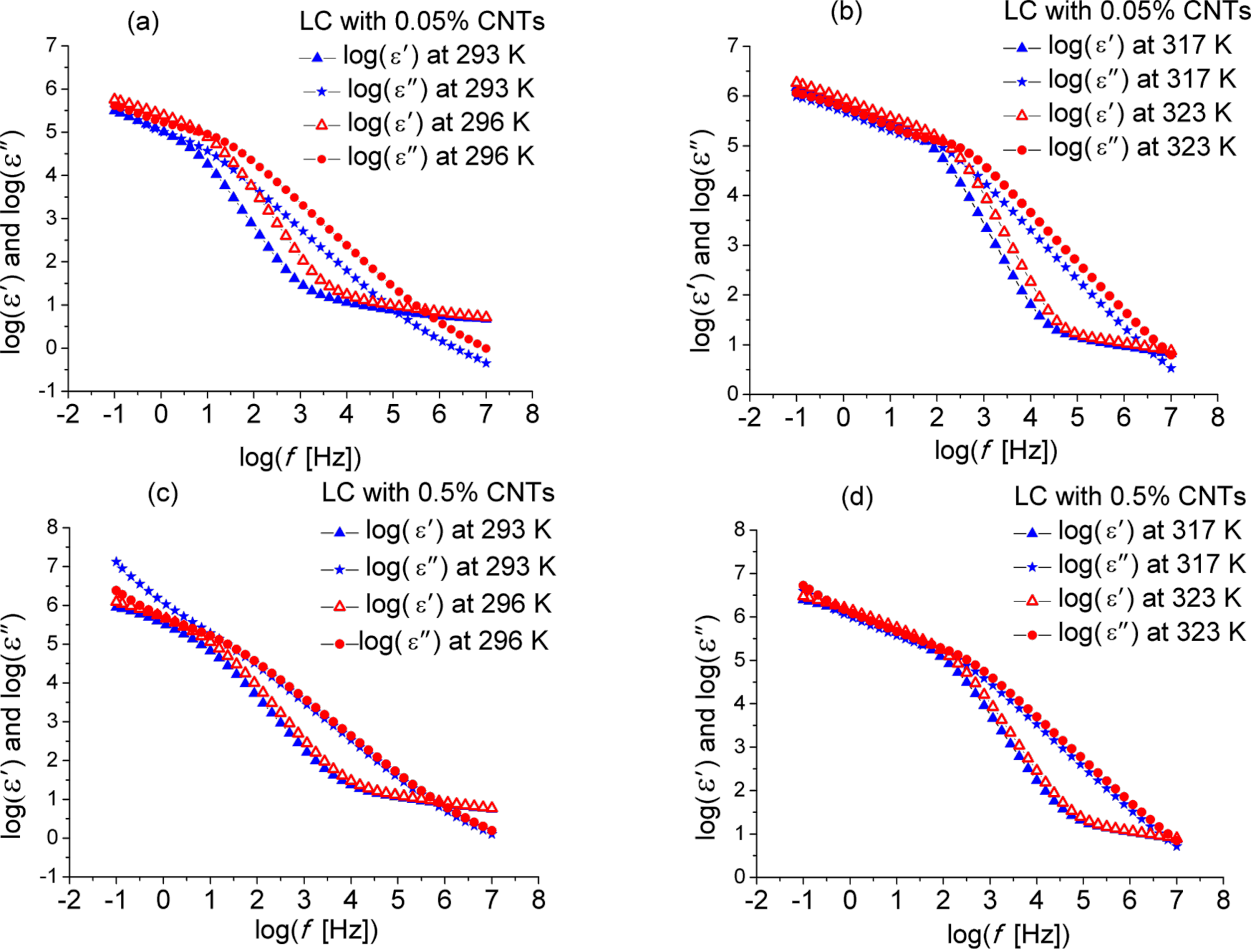
Permittivity and dielectric loss as functions of the frequency (logarithmic scale) at different constant temperatures for (a,b) ILC with 0.05% CNTs, and (c,d) ILC with 0.5% CNTs.

There are “jumps” in the characteristic relaxation time around certain temperatures such as 293–296 K for the CNT-doped samples and 314.15–323.15 K for the pure ILC and the 0.05% CNT doped sample (Figure 7). These jumps can be attributed either to the fitting procedure or to one or more phase transitions. The isotropic–SmA transition is found in the range of 314–323 K. In order to study this phenomenon, the permittivity and dielectric loss were plotted as functions of the frequency in the abovementioned temperature ranges. These curves are shown in Figure 8 and Figure 9. The ILC permittivity as a function of the frequency has three distinct slopes, one between 10^−1^ and 10^2^ Hz, one between 10^2^ and 10^4^ Hz and the third one between 10^4^ and 10^7^ Hz. This type of dependence also occurs for the CNT doping concentrations of 0.05% and 0.5%.

The dielectric loss of the ILC can be divided in two regions, between 10^−1^ and 10^2^ Hz and between 2·10^2^ and 10^7^ Hz (Figure 8). At lower temperatures, for the CNT- doped samples (Figure 9a,b), the straight lines with the greater slope are seen over a wider frequency range (more evident in Figure 9c, at 293 K). In the dielectric loss spectra, presented in Figure 8 and Figure 9, a dipolar relaxation process was identified in the range of 10^2^–10^3^ Hz, with only the descendent slope being observed. The Havriliak–Negami fitting function (Equation 1) are centered on this process. In the fitting procedure the exponent β was set to 1, and the other parameters were left optimized by fitting the HN function to the experimental data. It was found that the permittivity and the dielectric loss values increase with the temperature for pure ILC and for all CNT-doped samples.

**Figure 10:**
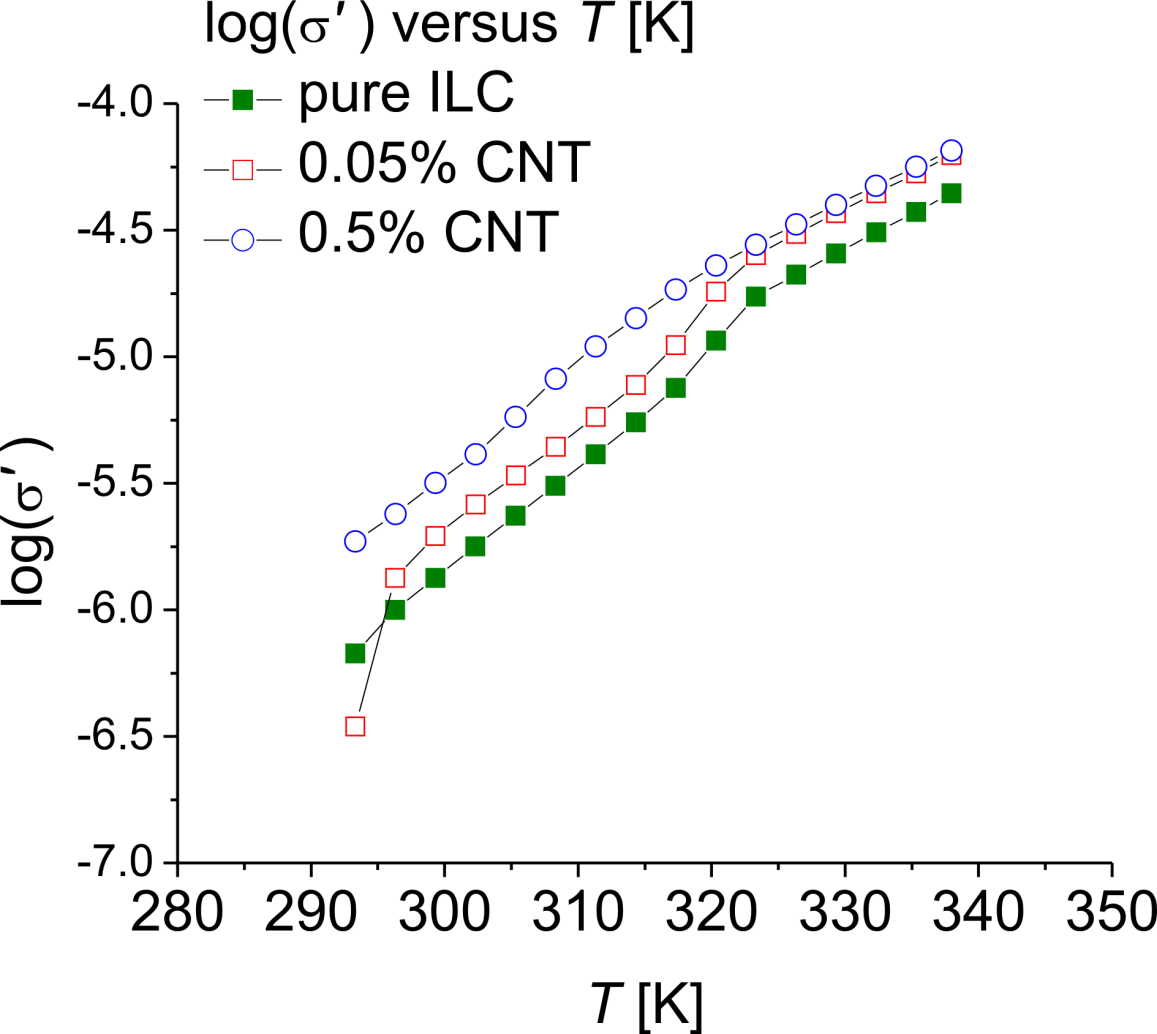
Real part of the conductivity versus temperature for pure ILC and ILC doped with CNTs, at 10 kHz.

The permittivity and the dielectric loss have high values in the LF domain, both for the pure and the doped-ILC. The high values in the range of 10^6^–10^8^ Hz are due to the presence of free ions. Figure 10 shows the variation of the real part of the conductivity as a function of the temperature for the ILC and the CNT-doped ILC. A linear dependency is observed, the change of the slope being attributed to the different phases. This behavior is more clearly observed for the ILC (solid squares) and the ILC doped with 0.05% CNTs (open squares). The conductivity increases with the CNT concentration.

**Figure 11:**
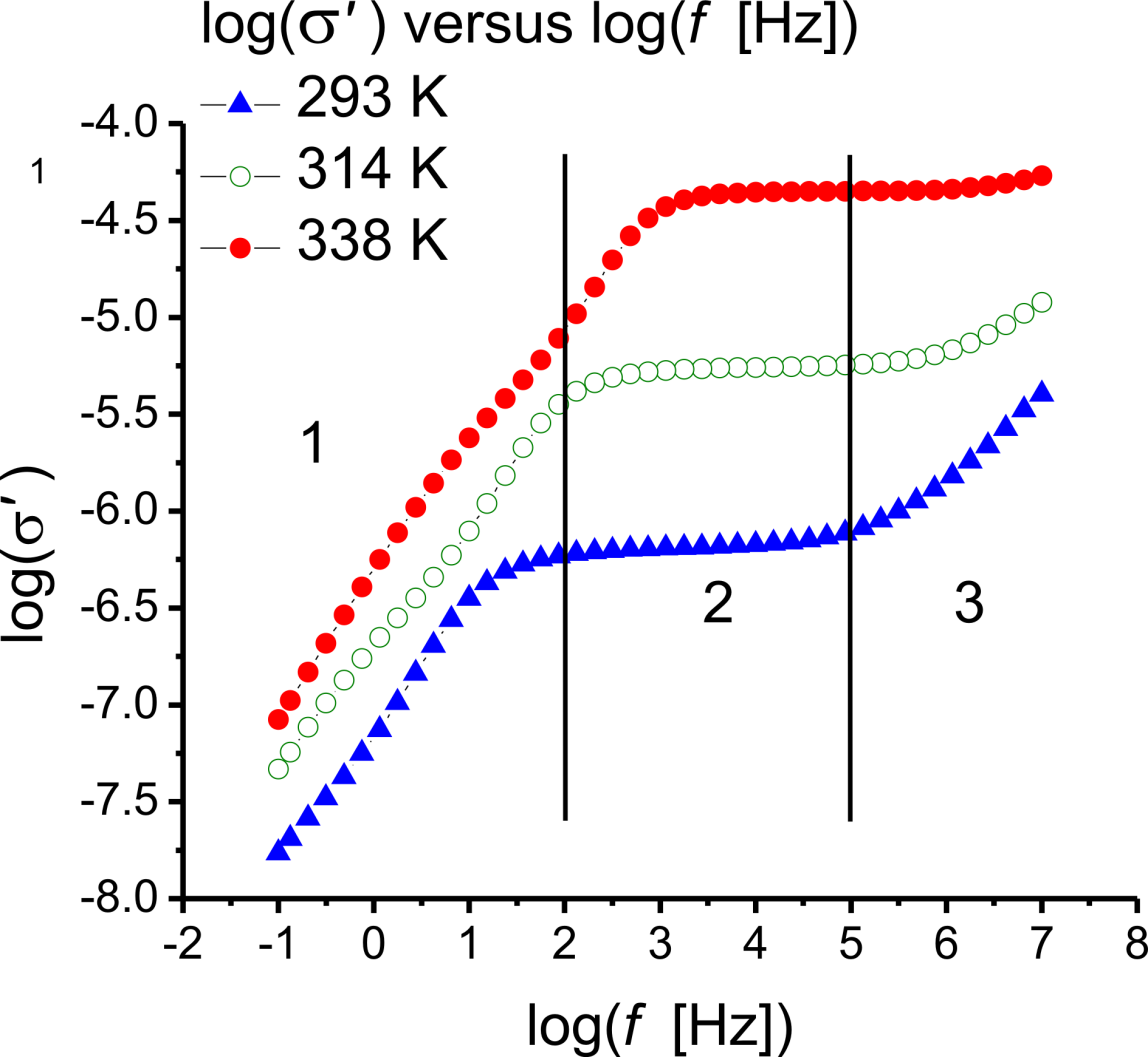
Real part of the conductivity as a function of the frequency (logarithmic scale) for the pure ILC, at three different constant temperatures. Region 1: low and very low frequency; region 2: medium frequency; region 3: high frequency.

**Figure 12:**
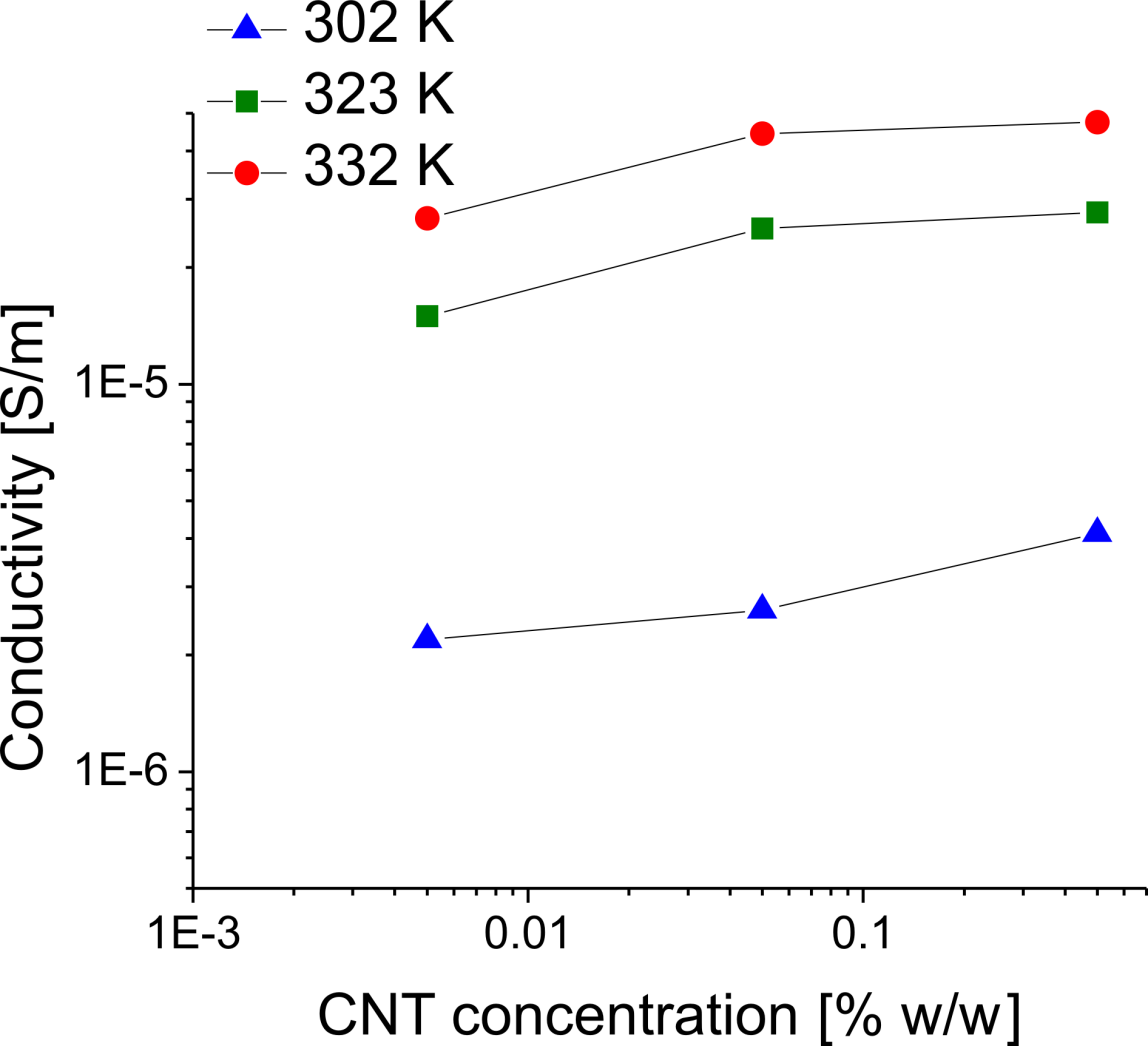
Conductivity as a function of the CNT concentration at three constant temperatures.

Figure 11 shows the real part of the conductivity of the pure ILC as a function of the frequency (logarithmic scale), at three different constant temperatures. An increase of the conductivity with CNT concentration and temperature is also observed as presented in Figure 12.

### Ionic conductibility

Two major polarizing mechanisms can be studied by means of DS: a) polarization due to orientation of permanent molecular electrical dipoles and b) polarization due to the movement of mobile charge carriers. Due to the presence of mobile charge carriers in LCs, a supplementary induced polarization needs to be considered when interpreting experimental data, namely the electrode polarization, resulting from charge accumulation at the electrode/sample interface.

Experimental data can be presented either by means of the complex dielectric function or of electric conductivity. The AC conductivity σ*(ω) is a complex function:

[3]
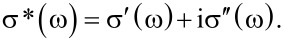


In agreement with Maxwell’s equations, a direct general relationship can be established between the electrical conductivity and the dielectric permittivity of a medium. For a sinusoidal electric field Equation 3 becomes:

[4]
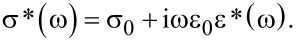


The real and the imaginary parts are σ′(ω) = σ_0_ + ωε_0_ε″(ω) and σ″(ω) = ωε_0_ε′(ω), respectively.

As shown in Figure 11, at medium frequencies (10^3^–10^5^ Hz), the ac conductivity and permittivity spectra are controlled by ion movements in the bulk of the electrolyte. At low frequencies (10^−1^–10^3^ Hz), approximately region 1 in Figure 11, the behavior is controlled by “electrode polarization” effects. Thus, the electric conductivity decreases significantly when the frequency decreases. In the frequency range below 100 MHz, the ionic conductivity spectra obey the Jonscher power law [52–53]:

[5]
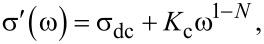


where 0 < *N* ≤ 1 and σ_dc_ is the dc conductivity (usually 0.1 ≤ *N* ≤ 0.4). The parameter σ_dc_ is obtained from the electrical conductivity spectra by extrapolation to the ω→0 limit.

For samples with ionic carriers, obtaining the conductivity values, σ_dc_, is not trivial [38,54–55]. In this case the effects of the electrode polarization and of the ionic conductivity overlap at medium and low frequencies in the conductivity spectra (Figure 11). The electric conductivity is σ_dc_ = *q*·μ·*n*, where *q* is the electrical charge (in C), *n* is the concentration (in cm^−3^), and μ is the mobility (in cm^2^·s^−1^·V^−1^).

The bulk electrical ionic conductivity σ_dc_ obeys the Arrhenius law [5]:

[6]
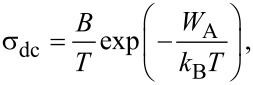


where *T* is the absolute temperature, *B* is a pre-exponential constant of the material and *W*_A_ is a constant associated with the activation energy. Usually, the activation energy includes the energy terms for the formation and migration of ions.

The experimental results showed that the real part of the permittivity, ε′, representing the dielectric conductivity, increases with the increase of CNT concentration; the same behavior was seen for the imaginary part of the permittivity, ε″, representing the dielectric loss. The permittivity being linked to dipole movements, an increase of the CNT concentration will lead to possible sample heating. Moreover, the ionic conductivity, σ, increases with CNT concentration (Figure 12). In this way, additional heating results from an increase of the CNT concentration, this process being frequency dependent.

## Conclusion

A new ILC based on bisimidazolium salt with a spacer of ten carbon atoms and octyl tails and dodecyl sulfate as counterion was synthesized. The SmA phase was unambiguously assigned based on polarized optical microscopy observations and XRD studies while the corresponding phase transition enthalpies were obtained by DSC. The ILC was doped with CNT in concentration of 0.05% w/w and 0.5% w/w. The dielectric spectra were recorded in the frequency range from 10^−1^ to 10^7^ Hz. The dependence of the dielectric constant and electric energy loss on frequency and temperature was studied. The activation energy was determined and the characteristic time was obtained by fitting the spectra of the dielectric loss with the Havriliak–Negami function. The study of the dielectric spectra leads to the following observations: (1) The study of the dielectric permittivity and electric conductivity spectra is complex, due to the superposition of ionic conductivity effect and dipolar relaxation specific to LC. Ionic conductivity is dominant and its effects are indirectly seen through the electrode polarization (EP) effect. (2) The very high dielectric permittivity values and the decrease of the electric conductivity at low frequencies confirm the presence of EP. (3) The doping with CNTs results in an increase of the conductivity. (4) Relaxation times decrease with CNT concentration. (5) In the lower temperature range, the presence of CNTs leads to a decrease of the activation energy while for higher temperatures, the activation energies are very similar for the pure ILC and the doped samples. As expected, the activation energies are lower at higher temperatures.

These preliminary studies revealed an interesting influence of the CNT concentration on the dielectric behavior of the bisimidazolium salt. This investigation will be extended to new ionic liquid crystals with a large range of doping concentration in order to complete the existing image on these aspects. Special attention will be paid to low-frequency studies of the effects related to very low CNT concentration.

## Experimental

### Characterization methods

All chemicals were used as supplied. C, H, N analyses were carried out with an EuroEA 3300 instrument. IR spectra were recorded on a Bruker spectrophotometer using KBr discs or by using a Jasco FTIR 4200 spectrophotometer coupled to an ATR PIKE GladiATR device. ^1^H and ^13^C NMR spectra were recorded on a Bruker spectrometer operating at 500 MHz, using CDCl_3_ as solvent. ^1^H chemical shifts were referenced to the solvent peak position, δ = 7.26 ppm. The phase assignment for the bisimidazolium salt was evaluated by polarizing optical light microscopy (POM) [56–57], placed on untreated glass slides, using a Nikon 50iPol microscope equipped with a Linkam THMS600 hot stage and TMS94 control processor. Temperatures and enthalpies of transitions were recorded by using differential scanning calorimetry (DSC) technique employing a Diamond DSC Perkin Elmer instrument. The bisimidazolium salt was studied at a scanning rate of 10 K/min after being encapsulated in an aluminum pan. Three heating–cooling cycles were performed for this sample.

### Synthesis of [bisC_8_ImC_10_][C_12_OSO_3_]_2_

A solution of sodium dodecyl sulfate (2.18 g, 7.5 mmol) in methanol (60 mL) was added dropwise to a solution of compound **2** (2 g, 3.0 mmol) in dichloromethane (50 mL). The mixture was stirred at room temperature for 1 h after which 100 mL of deionised water was added. The organic layer was separated and washed repeatedly with water until no reaction with silver nitrate for Br^−^ was noticed. The organic phase was dried over sodium sulfate followed by solvent removal with a rotary evaporator. The product was recrystallized twice from a mixture of dichloromethane and ethyl ether to yield an off-white waxy solid. Yield 75%, off-white waxy solid. Anal. calcd for C_56_H_110_N_4_O_8_S_2_: C, 65.20; H, 10.75; N, 5.43; found: C, 65.59; H, 11.03; N, 5.27; ^1^H NMR (500 MHz, CDCl_3_) δ 9.58 (s, 2H), 7.78 (s, 2H), 7.24 (s, 2H), 4.32–4.20 (m, 8H), 4.01 (t, 4H), 1.94-1.84 (t, 8H), 1.64 (t, 4H), 1.42–1.22 (m, 68H), 0.86 (t, 12H); ^13^C NMR (125 MHz, CDCl_3_) δ 136.9, 123.3, 121.6, 67.8, 49.9, 49.4, 31.9; 29.7, 29.6, 29.5, 29.4, 22.7, 14.1; IR (ATR, cm^−1^): 3136, 3109, 2957, 2919, 2851, 1568, 1467, 1379, 1225, 1169, 1063, 1044, 1005, 932, 792, 723, 623, 580.

### Preparation of the mixtures of LC doped with CNTs

Single-walled CNTs (Aldrich code 519308) with a diameter between 1.2 and 5 nm were employed in this study. Two CNT/[bisC_8_ImC_10_][C_12_H_25_OSO_3_]_2_ mixtures with different amounts of CNTs (0.5% w/w and 0.05% w/w) were prepared for dielectric measurements by consecutive dilution of an initial sample containing 2% CNTs. The starting sample was prepared by dissolving first the bisimidazolium salt (0.2 g) in a minimum volume of dichloromethane (1 mL) followed by addition of CNT (0.004 g) [58]. The resulting mixture was sonicated for at least 60 min followed by the removal of the solvent, drying in vacuum and cooling at 0 °C. All samples were kept at 0 °C before the physical measurements. Prior to dielectric measurements, the samples were heated to 50 °C and sonicated for at least 15 min to ensure an homogeneous dispersion of CNTs in the ionic liquid crystalline sample.

### X-ray diffraction

The X-ray diffraction measurements were made on a D8 Advance diffractometer (Bruker AXS GmbH, Germany), in parallel beam setting, with monochromatized Cu Kα_1_ radiation (λ = 1.5406 Å), scintillation detector, and horizontal sample stage. The measurements were performed in symmetric (θ–θ) geometry in the 2θ range from 1.5 to 30° in steps of 0.02°, with measuring times per step in the range of 5–40 s. The sample was deposited on a Si(100) plate, heated to the isotropic state and then cooled down to room temperature prior to data acquisition

### Dielectric spectroscopy

The dielectric spectroscopy measurements were performed using a broadband dielectric spectrometer, NOVOCONTROL, with an Alpha-A high-performance frequency analyzer in the LF domain (0.01 to 10^7^ Hz), equipped with WinDETA software. The temperature was controlled within 0.2 K, at a constant ac voltage of 0.5 V.
